# Supplementary material for: Chromothripsis during telomere crisis is independent of NHEJ, and consistent with a replicative origin
Source: Genome Res. 2019 May;29(5):737–49. doi: 10.1101/gr.240705.118 (PMC6499312; doi:10.1101/gr.240705.118)
Supplement: Supplemental Material [file supp_gr.240705.118_Supplemental_file_1.zip › contigs/annotated_contigs/DB73/contig.2.DB73_length_287_mean_cov_20.4599303136.docx]

**DB73_length_287_mean_cov_20.4599303136**

```
GGGGGGGGGGGGGGGGGGGGGGGGGGGGGGGGGGGGGGGGGGGGGGGGGGGGGGT|TTTTTCAAGCAGAAGACGGCATACGAGATCATA
 >chr2:33141359-33141414 + E=1e-15
GCCGGTGACTGGAGTTCAGACGTGTGCTCTTCCGATCT|CCACTAGCCCCTGAGGTCTACCAAAGGTAGCACATGGGGAGTTCCCCTAA
                                       >chr1:50286738-50286844 + E=2e-52
AAAATAGGCTGATTGTGCGGTCCAGTATGCTTTTAAAAGGAATAGGTACCATGAAG|AGATCGGAAGAGCGTCGTGTAGGGAAAGAGTG

TGCCTCTATGTGTAGATCTCGGT
```
